# Supplementary material for: Effects of bacterial composition and aquatic habitat metabolites on malaria vector larval availability in irrigated and non-irrigated sites of Homa Bay county, western Kenya
Source: PLoS One. 2023 Jun 2;18(6):e0286509. doi: 10.1371/journal.pone.0286509 (PMC10237499; doi:10.1371/journal.pone.0286509)
Supplement: S1 File — (DOCX) [file pone.0286509.s001.docx]

**SUPPORTING INFORMATION FILE**

**Table SIF 1:** Identity matrix table showing the frequency of relationship or association between isolated bacteria and GenBank accession numbers.

| **GENBANK ACCESSION** | **MC9** | **NA7** | **NA2A** | **EMB9A** | **NA9** | **NA4A** | **NA10** | **MC2** | **NA3** | **NA5AA** | **NA10A** |
| --- | --- | --- | --- | --- | --- | --- | --- | --- | --- | --- | --- |
| OX245674 (*Citrobacter* sp. Marseille-Q6884) | 100.00 | 57.05 | 59.34 | 27.14 | 30.77 | 32.08 | 31.75 | 32.94 | 32.94 | 32.35 | 32.35 |
| LC588555 (*Bacillus siralis* J35TS1) | 57.05 | 99.91 | 70.23 | 27.04 | 28.60 | 31.04 | 32.83 | 33.48 | 33.43 | 32.78 | 32.60 |
| OX216966 (*Paenibacillus dendritiformis* ) | 59.56 | 70.65 | 99.55 | 25.83 | 28.27 | 31.46 | 31.51 | 32.09 | 32.09 | 31.34 | 31.34 |
| CP102379 (*Escherichia coli* strain BM28 lys) | 27.14 | 27.04 | 25.63 | 99.70 | 57.31 | 56.80 | 70.11 | 56.90 | 56.90 | 55.21 | 54.96 |
| JQ265468 (Uncultured bacterium clone T2C182) | 27.14 | 27.04 | 25.63 | 100.00 | 57.31 | 56.55 | 70.11 | 56.90 | 56.90 | 55.21 | 54.96 |
| OP501807 (*Enterococcus gallinarum* strain 10 A) | 30.77 | 28.60 | 28.57 | 57.44 | 99.78 | 81.03 | 86.10 | 86.61 | 86.61 | 83.48 | 84.58 |
| OP402856 (*Staphylococcus arlettae* strain Dg-E8) | 32.08 | 31.04 | 31.46 | 56.55 | 80.92 | 100.00 | 87.72 | 91.11 | 91.28 | 89.26 | 89.02 |
| MH746088 (*Bacillus siralis* strain PA02) | 31.75 | 32.39 | 31.51 | 70.11 | 86.54 | 88.46 | 99.26 | 91.12 | 91.12 | 92.01 | 91.72 |
| OK570087 (*Robertmurraya siralis* strain C3) | 31.75 | 32.39 | 31.51 | 70.11 | 86.54 | 88.46 | 99.26 | 91.12 | 91.12 | 92.01 | 91.72 |
| ON430535 (*Bacillus cereus* strain CUMB AR-04) | 32.94 | 33.48 | 32.09 | 56.90 | 86.50 | 91.11 | 90.39 | 100.00 | 100.00 | 92.20 | 93.26 |
| ON860698 (*Bacillus cereus* strain PSR21) | 32.94 | 33.43 | 32.09 | 56.90 | 86.50 | 91.28 | 90.39 | 100.00 | 100.00 | 92.23 | 93.26 |
| OP435764 (*Bacillus tequilensis* strain A37) | 32.35 | 32.60 | 30.97 | 54.96 | 84.47 | 89.02 | 90.98 | 93.26 | 93.26 | 99.81 | 100.00 |
| KC441741 (*Bacillus subtilis* strain B59) | 32.40 | 32.87 | 31.02 | 55.02 | 83.43 | 88.87 | 91.05 | 92.47 | 92.47 | 99.77 | 99.86 |
| OP521932 (*Bacillus stercoris* strain ML-2) | 32.54 | 32.93 | 31.16 | 55.21 | 83.59 | 89.26 | 91.27 | 92.37 | 92.40 | 99.84 | 99.81 |
| OP482166 (*Bacillus mojavensis* strain M) | 32.15 | 32.83 | 30.78 | 54.96 | 83.37 | 89.12 | 90.98 | 92.19 | 92.23 | 99.49 | 99.62 |
| OP218479 (*Bacillus mojavensis* strain NR 112725) | 32.35 | 32.92 | 30.97 | 55.21 | 84.35 | 88.81 | 91.27 | 93.28 | 93.28 | 99.44 | 99.42 |
| OP514801 (*Escherichia coli* strain ASBY05) | 31.95 | 26.08 | 28.06 | 32.23 | 38.95 | 39.11 | 38.06 | 39.79 | 39.79 | 39.27 | 39.53 |
| OP793848 (*Exiguobacterium profundum* strain T5) | 30.56 | 32.38 | 29.46 | 55.58 | 78.68 | 80.84 | 83.28 | 84.51 | 83.42 | 84.01 | 85.16 |
| OP263689 (*Exiguobacterium profundum* strain H-1) | 30.56 | 32.38 | 29.46 | 55.58 | 78.68 | 80.84 | 83.28 | 84.51 | 83.42 | 84.01 | 85.16 |
| KF732994 (*Bacillus subtilis* strain PMM8) | 33.06 | 26.59 | 31.47 | 31.67 | 38.20 | 38.86 | 39.66 | 38.81 | 38.76 | 39.76 | 40.00 |
| OP554433 (*Bacillus velezensis* strain r22) | 33.06 | 26.96 | 31.47 | 31.67 | 38.20 | 38.94 | 39.66 | 38.90 | 38.84 | 39.64 | 39.80 |
| ON999044 (*Bacillus inaquosorum* strain GZCB-3) | 33.06 | 29.12 | 31.47 | 31.45 | 39.23 | 38.82 | 40.06 | 39.41 | 39.41 | 39.78 | 39.96 |
| OP115492 (*Bacillus aerius* strain GS26) | 32.21 | 31.71 | 30.83 | 56.80 | 85.38 | 88.62 | 92.75 | 91.89 | 91.89 | 93.51 | 94.22 |

| **GENBANK ACCESSION** | **NA10B** | **NA4BB** | **NA12AA** | **MC5B** | **NA5B** | **EMB9** | **MC1A** | **NABAA** | **NABA** | **EMB6** |
| --- | --- | --- | --- | --- | --- | --- | --- | --- | --- | --- |
| OX245674 (*Citrobacter* sp. Marseille-Q6884) | 32.35 | 32.54 | 32.25 | 31.42 | 31.28 | 31.95 | 30.75 | 30.56 | 30.56 | 32.21 |
| LC588555 (*Bacillus siralis* J35TS1) | 32.83 | 32.70 | 32.90 | 23.73 | 23.54 | 26.08 | 32.68 | 32.30 | 32.55 | 32.18 |
| OX216966 (*Paenibacillus dendritiformis* ) | 31.34 | 31.53 | 31.25 | 28.45 | 28.51 | 28.32 | 30.02 | 29.83 | 29.83 | 31.40 |
| CP102379 (*Escherichia coli* strain BM28 lys) | 54.96 | 55.21 | 55.09 | 31.31 | 31.38 | 32.23 | 55.34 | 55.34 | 55.34 | 56.80 |
| JQ265468 (Uncultured bacterium clone T2C182) | 54.96 | 55.21 | 55.09 | 31.31 | 31.38 | 32.23 | 55.34 | 55.34 | 55.34 | 56.80 |
| OP501807 (*Enterococcus gallinarum* strain 10 A) | 83.48 | 83.89 | 83.59 | 36.36 | 35.91 | 39.08 | 78.35 | 78.57 | 78.57 | 86.59 |
| OP402856 (*Staphylococcus arlettae* strain Dg-E8) | 88.83 | 88.25 | 89.03 | 38.18 | 38.43 | 39.11 | 80.50 | 80.06 | 80.76 | 90.48 |
| MH746088 (*Bacillus siralis* strain PA02) | 91.72 | 92.01 | 91.86 | 37.47 | 37.59 | 38.06 | 82.54 | 83.28 | 83.28 | 93.49 |
| OK570087 (*Robertmurraya siralis* strain C3) | 91.72 | 92.01 | 91.86 | 37.47 | 37.59 | 38.06 | 82.54 | 83.28 | 83.28 | 93.49 |
| ON430535 (*Bacillus cereus* strain CUMB AR-04) | 92.42 | 92.83 | 92.28 | 38.42 | 37.66 | 39.79 | 84.01 | 83.79 | 84.92 | 93.31 |
| ON860698 (*Bacillus cereus* strain PSR21) | 92.42 | 92.83 | 92.31 | 38.42 | 37.61 | 39.79 | 82.93 | 83.06 | 84.46 | 93.31 |
| OP435764 (*Bacillus tequilensis* strain A37) | 99.81 | 99.80 | 99.52 | 38.42 | 38.51 | 39.53 | 84.59 | 85.07 | 85.07 | 94.60 |
| KC441741 (*Bacillus subtilis* strain B59) | 99.86 | 99.65 | 99.49 | 38.27 | 38.24 | 39.33 | 82.86 | 83.32 | 83.32 | 94.66 |
| OP521932 (*Bacillus stercoris* strain ML-2) | 99.82 | 99.80 | 99.58 | 38.22 | 38.31 | 39.27 | 83.52 | 83.39 | 83.93 | 95.26 |
| OP482166 (*Bacillus mojavensis* strain M) | 99.44 | 99.40 | 99.92 | 38.42 | 38.38 | 39.53 | 83.45 | 83.18 | 83.87 | 94.57 |
| OP218479 (*Bacillus mojavensis* strain NR 112725) | 99.43 | 99.80 | 99.91 | 38.22 | 38.29 | 39.27 | 84.89 | 85.10 | 85.35 | 94.55 |
| OP514801 (*Escherichia coli* strain ASBY05) | 39.27 | 39.27 | 39.40 | 100.00 | 100.00 | 100.00 | 38.32 | 38.45 | 38.45 | 39.63 |
| OP793848 (*Exiguobacterium profundum* strain T5) | 83.36 | 84.66 | 84.04 | 38.25 | 37.21 | 38.58 | 98.17 | 99.65 | 99.84 | 87.35 |
| OP263689 (*Exiguobacterium profundum* strain H-1) | 83.36 | 84.66 | 84.04 | 38.25 | 37.21 | 38.58 | 99.54 | 99.82 | 99.84 | 87.35 |
| KF732994 (*Bacillus subtilis* strain PMM8) | 39.66 | 39.93 | 39.74 | 81.74 | 82.55 | 81.90 | 37.70 | 38.28 | 39.02 | 40.68 |
| OP554433 (*Bacillus velezensis* strain r22) | 39.51 | 39.93 | 39.79 | 81.74 | 82.55 | 81.90 | 38.25 | 38.38 | 39.10 | 40.51 |
| ON999044 (*Bacillus inaquosorum* strain GZCB-3) | 39.81 | 40.00 | 40.06 | 80.49 | 80.44 | 81.60 | 40.00 | 40.28 | 40.09 | 40.59 |
| OP115492 (*Bacillus aerius* strain GS26) | 93.31 | 94.29 | 93.21 | 39.46 | 40.00 | 39.63 | 84.55 | 84.96 | 84.96 | 100.00 |

| **GENBANK ACCESSION** | **NA2BA** | **NA12B** | **NA4BA** | **NASAB** | **NA6B** | **NA12AB** | **NA2B** | **NA7C** | **NA11A** | **EMB6A** |
| --- | --- | --- | --- | --- | --- | --- | --- | --- | --- | --- |
| OX245674 (*Citrobacter* sp. Marseille-Q6884) | 32.85 | 33.06 | 33.06 | 33.26 | 33.06 | 32.96 | 33.06 | 34.59 | 33.13 | 32.21 |
| LC588555 (*Bacillus siralis* J35TS1) | 26.59 | 26.96 | 26.96 | 29.12 | 26.96 | 27.04 | 28.94 | 30.35 | 26.37 | 31.71 |
| OX216966 (*Paenibacillus dendritiformis* ) | 31.47 | 31.66 | 31.66 | 31.57 | 31.66 | 31.76 | 31.27 | 32.16 | 31.73 | 31.20 |
| CP102379 (*Escherichia coli* strain BM28 lys) | 31.45 | 31.67 | 31.67 | 31.41 | 31.67 | 31.78 | 31.45 | 31.76 | 31.78 | 56.80 |
| JQ265468 (Uncultured bacterium clone T2C182) | 31.45 | 31.67 | 31.67 | 31.41 | 31.67 | 31.78 | 31.45 | 31.76 | 31.78 | 56.80 |
| OP501807 (*Enterococcus gallinarum* strain 10 A) | 38.30 | 38.30 | 38.30 | 40.52 | 38.30 | 38.30 | 39.34 | 42.97 | 38.98 | 85.49 |
| OP402856 (*Staphylococcus arlettae* strain Dg-E8) | 39.03 | 39.08 | 39.00 | 39.09 | 38.82 | 39.03 | 38.45 | 40.64 | 39.25 | 88.62 |
| MH746088 (*Bacillus siralis* strain PA02) | 39.66 | 39.66 | 39.66 | 40.13 | 39.66 | 39.66 | 40.06 | 41.95 | 42.61 | 93.49 |
| OK570087 (*Robertmurraya siralis* strain C3) | 39.66 | 39.66 | 39.66 | 40.13 | 39.66 | 39.66 | 40.06 | 41.95 | 42.61 | 93.49 |
| ON430535 (*Bacillus cereus* strain CUMB AR-04) | 38.98 | 39.03 | 38.95 | 39.72 | 38.78 | 38.98 | 39.04 | 42.37 | 40.03 | 91.89 |
| ON860698 (*Bacillus cereus* strain PSR21) | 38.93 | 38.98 | 38.90 | 39.72 | 38.73 | 38.93 | 39.04 | 42.37 | 40.03 | 91.89 |
| OP435764 (*Bacillus tequilensis* strain A37) | 40.00 | 39.80 | 39.93 | 40.40 | 39.80 | 39.88 | 39.59 | 41.74 | 41.18 | 94.22 |
| KC441741 (*Bacillus subtilis* strain B59) | 39.87 | 39.55 | 39.65 | 40.29 | 39.55 | 39.63 | 39.49 | 41.61 | 40.93 | 93.36 |
| OP521932 (*Bacillus stercoris* strain ML-2) | 39.93 | 39.77 | 39.70 | 40.32 | 39.65 | 39.72 | 39.41 | 41.37 | 40.89 | 93.51 |
| OP482166 (*Bacillus mojavensis* strain M) | 40.00 | 40.00 | 39.93 | 40.71 | 39.87 | 39.95 | 39.78 | 41.77 | 41.23 | 93.10 |
| OP218479 (*Bacillus mojavensis* strain NR 112725) | 39.79 | 39.97 | 39.90 | 40.51 | 39.84 | 39.92 | 39.60 | 41.15 | 41.14 | 94.05 |
| OP514801 (*Escherichia coli* strain ASBY05) | 81.60 | 81.90 | 81.90 | 81.90 | 81.90 | 81.90 | 81.60 | 82.79 | 82.20 | 39.63 |
| OP793848 (*Exiguobacterium profundum* strain T5) | 38.19 | 38.63 | 38.51 | 40.56 | 38.45 | 38.48 | 39.82 | 40.24 | 38.91 | 85.16 |
| OP263689 (*Exiguobacterium profundum* strain H-1) | 37.86 | 38.47 | 38.51 | 40.56 | 38.45 | 38.48 | 39.82 | 40.24 | 38.91 | 85.16 |
| KF732994 (*Bacillus subtilis* strain PMM8) | 99.50 | 100.00 | 100.00 | 100.00 | 100.00 | 99.91 | 99.34 | 95.26 | 90.32 | 41.18 |
| OP554433 (*Bacillus velezensis* strain r22) | 99.62 | 100.00 | 100.00 | 100.00 | 100.00 | 99.91 | 99.37 | 95.58 | 90.57 | 41.18 |
| ON999044 (*Bacillus inaquosorum* strain GZCB-3) | 99.56 | 99.79 | 99.79 | 99.78 | 99.79 | 99.69 | 99.59 | 93.10 | 89.81 | 41.25 |
| OP115492 (*Bacillus aerius* strain GS26) | 41.00 | 41.18 | 41.18 | 41.38 | 41.18 | 41.27 | 41.05 | 42.23 | 42.60 | 100.00 |
