# Supplementary material for: Effects of bacterial composition and aquatic habitat metabolites on malaria vector larval availability in irrigated and non-irrigated sites of Homa Bay county, western Kenya
Source: PLoS One. 2023 Jun 2;18(6):e0286509. doi: 10.1371/journal.pone.0286509 (PMC10237499; doi:10.1371/journal.pone.0286509)
Supplement: S1 Table — (DOCX) [file pone.0286509.s002.docx]

**SUPPORTING INFORMATION FILE**

**Table S1.** Blast results of samples collected from the field as identified by sequencing

| **Area classification** | **Sample identity** | **Blast results** | **Genus** |
| --- | --- | --- | --- |
| Irrigated | MC9 | *Citrobacter sp*. Marseille-Q6884 | *Citrobacter* |
| Irrigated | NA2A | *Paenibacillus dendritiformis* | *Paenibacillus* |
| Irrigated | EMB9A | Uncultured bacterium clone T2C182 | *Escherichia* |
| Irrigated | NA9 | *Enterococcus gallinarum* strain 10 A | *Enterococcus* |
| Irrigated | NA10 | *Bacillus siralis* strain PA02 | *Bacillus* |
| Irrigated | MC2 | *Bacillus cereus* strain CUMB AR-04 | *Bacillus* |
| Irrigated | NA5AA | *Bacillus stercoris* strain ML-2 | *Bacillus* |
| Irrigated | NA10A | *Bacillus tequilensis* strain A37 | *Bacillus* |
| Irrigated | NA10B | *Bacillus subtilis* strain B59 | *Bacillus* |
| Irrigated | MC5B, NA5B, EMB9 | *Escherichia coli* strain ASBY05 | *Escherichia* |
| Irrigated | MC1A, NA8AA, NA8A | *Exiguobacterium profundum* strain H-1 | *Exiguobacterium* |
| Irrigated | EMB6 | *Bacillus aerius* strain GS26 | *Bacillus* |
| Irrigated | NA2BA | *Bacillus velezensis* strain r22 | *Bacillus* |
| Irrigated | NA5AB, NA6B | *Bacillus subtilis* strain PMM8 | *Bacillus* |
| Irrigated | NA2B | *Bacillus inaquosorum* strain GZCB-3 | *Bacillus* |
| Irrigated | EMB6A | *Bacillus aerius* strain GS26 | *Bacillus* |
| Non-Irrigated | NA7 | *Bacillus siralis* J35TS1 | *Bacillus* |
| Non-Irrigated | NA4A | *Staphylococcus arlettae* strain Dg-E8 | *Staphylococcus* |
| Non-Irrigated | NA3 | *Bacillus cereus* strain CUMB AR-04 | *Bacillus* |
| Non-Irrigated | NA4BB | *Bacillus stercoris* strain ML-2 | *Bacillus* |
| Non-Irrigated | NA12AA | *Bacillus mojavensis* strain M | *Bacillus* |
| Non-Irrigated | NA12B, NA4BA, NA7C, NA11A | *Bacillus velezensis* strain r22 | *Bacillus* |
| Non-Irrigated | NA12AB | *Bacillus subtilis* strain PMM8 | *Bacillus* |
