# Supplementary material for: Effects of bacterial composition and aquatic habitat metabolites on malaria vector larval availability in irrigated and non-irrigated sites of Homa Bay county, western Kenya
Source: PLoS One. 2023 Jun 2;18(6):e0286509. doi: 10.1371/journal.pone.0286509 (PMC10237499; doi:10.1371/journal.pone.0286509)
Supplement: Attachment — Submitted filename: MS- for inputs.doc [file pone.0286509.s003.doc]

**Effects of bacterial composition and aquatic habitat metabolites on malaria vector larval availability in irrigated and non- irrigated sites of Homa Bay county, western Kenya**

Pauline Winnie Orondo^1*^, Kevin O. Ochwedo^2^, Harrysone Atieli^2^, Guiyun Yan^3^, Andrew K. Githeko^4^, Steven G. Nyanjom^1*^

**Author’s affiliations**

# ^1.^ Department of Biochemistry, Jomo Kenyatta University of Agriculture and Technology, Nairobi, Kenya

# ^2.^ International Center of Excellence for Malaria Research, Tom Mboya University, College of Maseno University, Homa Bay, Kenya

# ^3.^ Program in Public Health, College of Health Sciences, University of California at Irvine, Irvine, California, USA

# ^4^ Centre for Global Health Research, Kenya Medical Research Institute, Kisumu, Kenya

***Correspondence author**

Email addresses: Pauline Winnie Orondo: [paulineorondo@gmail.com](mailto:paulineorondo@gmail.com); Kevin O. Ochwedo: [kevinochwedo@gmail.com](mailto:kevinochwedo@gmail.com); Harrysone Atieli: [etemesi2012@yahoo.com](mailto:etemesi2012@yahoo.com); Guiyun Yan: [guiyuny@uci.edu](mailto:guiyuny@uci.edu); Andrew K. Githeko: [githeko@yahoo.com](mailto:githeko@yahoo.com); Steven G. Nyanjom: snyanjom@jkuat.ac.ke.

**Abstract**

Gravid *Anopheles* malaria vectors depend on both chemical and physical (including microbial) cues for selection of preferred habitats for oviposition. The purpose of this study was to assess the effects of bacterial composition and habitat metabolites on malaria vector larval availability in irrigated and non-irrigated potential larval sources. Water samples were collected from larval positive and negative habitats in the irrigated and non- irrigated areas of Homa Bay county. Bacteria cultured from the water samples were subjected to Matrix Assisted Laser Desorption Ionization-Time of Flight Mass Spectrometry (MALDI-TOF MS) for species identification. DNA was extracted from the colonies and polymerase chain reaction (PCR) and sequencing done. Finally, the metabolite composition of larval positive and negative habitats was determined. MALDI-TOF MS results revealed that *Bacillus* was the only genera identified from larval sources in non-irrigated zone. In the irrigated area, *Shigella* was the dominant genera (47%) while *Escherichia coli* was the abundant species (13/51). Of the sequenced isolates, 65% were *Bacillus*. Larvicidal isolates *Brevibacillus brevis*, *Bacillus subtilis*, and *Exiguobacterium profundum* were isolated and grouped with *Bacillus mojavensis*, *Bacillus tequilensis*, *Bacilus stercoris*, and *Brevibacillus agri*. Irrigated areas with larvae had reduced crude fat (0.01%) and protein content (0.13%) in comparison to those without larvae. In irrigated and non- irrigated areas, larval presence was evident in habitats with high total chlorophyll content (1.12 µg/g vs 0.81µg/g and 3.37 µg/g vs 0.82). Aquatic habitats with larvae in both irrigated and non-irrigated areas exhibited higher sugar concentration than habitats without larvae; however, when compared, non-irrigated areas with larvae had higher sugar concentration than similar habitats in irrigated areas. In addition, substantial concentrations of Manganese, Calcium, and Copper were found in aquatic habitats containing larvae in both irrigated and non-irrigated areas. These results allows for prospective examination as potential larvicidal or adulticidal agents and could be considered when designing potential vector control interventions

**Key words:** *Anopheles*, Microbial, Metabolites, Larval availability, Irrigated, Non-irrigated

**Introduction**

Malaria is a parasitic infection that results after an infective bite by *Anopheles* mosquito. These vectors are widely distributed within the tropics of Africa , Asia and central America . In Kenya, the main vectors have been observed to be complexes of *An. gambiae* and *An. funestus* which occur in sympatry over vast geographical regions . The distribution of these malaria vectors has been greatly linked to the aquatic habitat composition as the different species prefer specific habitats. In addition to the physical characteristics of the habitats there are known to be other chemical and biological cues that attract for oviposition of gravid mosquitoes . These oviposition sites have differing composition of both biotic and abiotic composition . The initial stages of a mosquito life-cycle is fully aquatic and this stage significantly affects their adult life in terms of survival, fitness and reproduction . The *Anopheles* mosquito has been observed to select for habitats that favor the survival and development of the immature .

Selection of oviposition sites for malaria vectors is dependent on a number of physical and chemical factors. Gravid malaria mosquitoes are attracted to water bodies by water vapour , as well as olfactory cues from con- specific larvae , microorganisms  and soil  associated with the sites. Chemical cues are known to originate from microbes , larval presence , decomposing matter which act as food for the larvae and predators in the habitats . Several chemical compounds have been reported to attract gravid mosquito for oviposition while others deter or have no effect on oviposition (Mwingira et al., 2020).

Microbial volatiles from larval habitats influence oviposition by mosquitoes . This is because micro-organisms act as food for the larvae. Studies have shown that algae may enhance *An gambiae* selection for a habitat for oviposition . As these bacterial volatiles act as attractants to different species , while reduced oviposition have been observed with reduction in bacterial concentration in aquatic habitats . Further studies are needed to confirm the oviposition attractant compounds for the development of mosquito traps and/ or in combination with larvicides for vector control. Additionally, volatiles produced by organic matter have been observed to attract gravid female *Anopheles* species for oviposition .

While habitat cues associated with the sites potentially attract mosquitoes over a longer distance, these have not been fully explored. Emergent and other vegetation may provide such potential cues, as grasses and other short vegetation often emerge from and surround natural larval habitats of malaria mosquitoes. Different vectors were observed to prefer certain habitat types to others, however, this has been observed to be changing over time. Originally it was understood that *An. funestus* bred mainly in more permanent marshes and swamps with tall vegetation . This notion is however changing as this species is now observed to inhabit habitats without vegetation . *An. gambiae* s.l. has been thought to prefer the edges of fresh slow moving temporary waters which are sunlit , however, recent studies have shown that these vectors can also infect more permanent habitats with vegetation cover like rice paddies . Vegetation also produces specific volatile compounds that can act as attractants or repellants. Attractants that have been isolated include a mixture of terpenoid and alcohol compounds .

Understanding the composition of oviposition sites is therefore essential as it is a potential area that can be explored in developing successful vector control strategies for insect- borne infectious diseases . Knowledge of the determinants that can be used as oviposition repellants or attractants is essential in this process. This study was undertaken to understand the chemical cues in aquatic larval mosquito habitats that attract or repel gravid female malaria vectors.

**Materials and methods**

**Study site**

The study was conducted in Homa Bay County, western Kenya, a semi-arid area situated along the shores of Lake Victoria (34.6 °E and 0.5 °S; 1,330 m above sea level). This area has been previously described by and is known to be malaria endemic . Briefly the study site was composed of an area with channel-based irrigation system that is used to assist in farming at the household level. This area is termed the irrigated area. The non- irrigated area is the area where irrigation channels do not exist to aid in farming.

**Sample Collection**

Water samples were collected between November 2021 to January 2022 from the irrigated and non- irrigated areas from larval infested and non- infested aquatic habitats. Larval positive habitats were those that were found to harbor malaria vectors while larval negative habitats were those that had no malaria vectors during the sampling time. The water samples were pooled into 12 bottle sets and used for microbial analysis. A minimum of 50 ml of water was collected from each aquatic habitat. Pooling was done on a monthly basis with a minimum volume of 1 liter per pool (**Table 1)**.

**Table 1:** Survey period, targeted areas for larval sources and larval availability.

| **Month/ Area** | **Irrigated** | | **Non-irrigated** | |
| --- | --- | --- | --- | --- |
| **November 2021** | Larval positive | Larval negative | Larval positive | Larval negative |
| **December 2021** | Larval positive | Larval negative | Larval positive | Larval negative |
| **January 2022** | Larval positive | Larval negative | Larval positive | Larval negative |

**Bacterial analysis**

**Bacterial culture**

Peptone water was added and incubated at 36 °C ± 2 °C for 16-20 hours. Bacteria was cultured in nutrient agar at 37 °Cfor 24 hours and single colonies plated on nutrient agar, Eosin Methylene Blue (EMB) agar and MacConkey agar at 37 °C for 24 hours. Individual colonies were then grown in nutrient broth for 24 hours and preserved in 50% glycerol for Matrix Assisted Laser Desorption Ionization-Time of Flight Mass Spectrometry (MALDI-TOF MS) and sequencing.

**Microbial Culture Analysis by MALDI-TOF MS**

Each bacterial colony identified from the culture was placed in duplicate on the MALDI-TOF plate target. The direct smear plus formic acid sample preparation method was utilized. Briefly, a colony was transferred from the agar surface to the target plate and was overlaid with 0.5 µl of 25% formic acid solution. After drying 0.5 ul of a saturated solution of alpha-cyano-4-hydroxycinnamic acid (10mg/ml) diluted in 250 ul solutions comprising of acetonitrile, HPLC grade water, and ethanol in a ratio of 3:3:3 and containing a final concentration of 3% of trifluoroacetic acid was added. The target plate was then analyzed using the MALDI-TOF spectrometry for bacterial identification against the SARAMIS database. Spectra range was recorded in linear mode within a mass range between 2000 to 20000 Daltons. The colonies were genotyped by MALDI-TOF MS and the plate read using Shimadzu software (Axima Confidence, Shimadzu, Japan).

**Polymerase chain reaction and sequencing**

Bacterial DNA was extracted from 31 selected colonies using the ISOLATE II Genomic DNA Kit (Bioline, London, UK) following the manufacturer’s instructions. Polymerase chain reaction was done using Go Taq Master Mix (Promega, Wiscosin, USA). A final PCR reaction volume of 25µl was prepared by addition of 12.5 µl of Go Taq master mix, 9.5µl nuclease free water, 0.3µl each of the forward and reverse primers and 2µl of the bacterial DNA template. The PCR reaction was set as follows in an ABI GeneAMP 9700 (Applied Biosystem, Foster City, CA, USA); 95°C for 5min, 35 cycles (94 °C for 30 sec,57 °C for 45 sec,72 °C for 45 sec), followed by final extension at 72 °C for 7 min. The quality of the amplicons was assessed through gel electrophoresis in 1.5% w/v agarose gel before purification and sequencing. Each of cleaned amplicons (using Exonuclease I and Shrimp Alkaline Phosphatase or ExoSAP-IT) were bi-directionally sequenced using BigDye® Terminator v3.1 Sequencing Standard kit on ABI PRISM® 3700 DNA Analyzer (Applied Biosystems, Foster City, CA, USA).

**Metabolite identification in irrigated and non-irrigated larval habitats**

Contents of crude protein, polyphenols, free radicals, fatty acids, fats and other minerals were determined according to Association of Official Analytical Chemists (AOAC) ® (2002) protocols .

**Crude Protein method**

10 ml of the sample was mixed with the catalyst (5g K_2_SO_4_, 0.5g CuSO_4_ and 15 ml concentrated H_2_SO_4_). The mixture was heated to blue color, cooled, transferred into a 100 ml volumetric flask and topped with distilled water. A blank digestion with the catalysts and acid was also made. 10 ml of diluted digest was transferred into a distilling flask and washed with 2ml of distilled water. The distillate was titrated using 0.02N - HCl to an orange color.

**Determination of total polyphenols**

Determination of total polyphenol was carried out using the method of with slight modification. 10 ml of sample was put in amber glass bottle and 50 ml of methanol added. Extraction was done for 3 hours in a shaker and the extract kept in the dark for 72 hours, filtered, then topped up to 50ml using methanol. The extract was them centrifuged for 10 min at 25 °C at 150 rpm. 2 ml of the supernatant was taken and filtered using 0.45 µl micro-filter, put in a test tube then 2 ml of 10% Folin-Ciocateu was added, vortexed and 4 ml of 0.7M sodium carbonate added then vortexed again. The extract was incubated for two hours to develop color and absorbance was read at 765 nm using gallic acid as a standard in a UV-Vis spectrophotometer (Shimadzu model UV – 1601 PC, Kyoto, Japan).

**Fatty acid analysis**

The fatty acid profile was determined by gas chromatography (GC). Extraction was done by a modification of the method. 100 ml of sample was mixed with 50ml of hexane and the mixture shaken overnight. 2 mg of the oil sample was refluxed with 5ml of 95% methanol- HCl for 1hour. The methyl esters were extracted with 3 portions of 5 ml hexane and then washed with 5 ml distilled water. The hexane layer was dried in vacuum rotary evaporator and the residue re-dissolved in 1 ml of hexane. Then 1µl was injected into the GC under split mode of 60 (Shimadzu GC-2010 equipped with auto sampler) with a capillary column, supelcowax 30m x 0.53 mm; injection temperature of 240 °C and detection temperature of 260 °C under aflame ionization detector. Identification of the fatty acid methyl esters was by comparison of retention times with standards and expressed as percentages of total methyl esters.

**Determination of the free radical scavenging activity**

The radical scavenging activities of the plant extracts against 2, 2-Diphenyl-1-picryl hydrazyl (DPPH) radical (Sigma-Aldrich) were determined by UV spectrophotometer at 517 nm . The following concentrations of the extracts were prepared, 0.01, 0.1, 1.0, 2.0 and 5 mg/ml in methanol (Analar grade). Vitamin C was used as the antioxidant standard with the same concentrations as the extract concentrations. 1 ml of the extract was placed in a test tube, and 3 ml of methanol added followed by 0.5 ml of 1 mM DPPH in methanol. A blank solution was prepared containing the same amount of methanol and DPPH. Methanol was used to zero the spectrophotometer and the absorbance was read at 517 nm after 5 minutes in UV-Vis spectrophotometer (Shimadzu model UV – 1601 PC, Kyoto, Japan).

**Mineral analysis**

100 ml of the water sample was put in a 250 ml beaker and placed on a hot plate following AOAC^®^ (1984) protocol . The water was heated and while about to boil, 2 ml of nitric acid- water mixture (50:50) and 10 ml HCL- water mixture (50:50) were added. The samples were evaporated until the mixture reduced to approximately 25 ml. The 25 ml residues were transferred into 100 ml volumetric flasks and filled to the mark with distilled water. The lead, cadmium, mercury and arsenic contents in the samples were determined using a Shimadzu Atomic Absorption Spectrophotometer, Model AA-7000.

**Lipid/ oil content analysis**

The fatty acid profile was determined by gas chromatography. The extraction of the lipids was done by a modification of the protocol. Samples of 100ml were mixed with 50 ml of hexane and the mixture shaken for 30 minutes and let to stand. The hexane layer was collected and the aqueous layer returned. The extraction was repeated one more time. The hexane fractions were combined and filtered using defatted cotton wool and anhydrous Na_2_SO_4_ to remove the water. The filtrate was concentrated at 40 °C and dried in the oven at 70 °C for 30 minutes.

**Determination of glucose, pectin, amylose and cellulose content**

This procedure followed AOAC (1996) protocol. 50 ml of the water was mixed with 50ml Acetonitrile. 2 ml of lead acetate was added. The solution was filtered in 5% anhydrous oxalate and micro-filtered. The individual sugars were analyzed using a high-performance liquid chromatography (HPLC) (Model LC-20A Series, Shimadzu Corp., Kyoto, Japan) fitted with a refractive index (RI) detector. The oven temperature was set at 30 °C, the flow rate was 0.5-1.0 ml/min, and the injection volume was 20 µl. The mobile phase used was acetonitrile and water in the volume ratio of 3:1. The sugars present were identified and their individual concentration calculated using the standards.

**Extraction of chlorophyll and carotenoid**

This followed protocol. 25ml of sample was added to 25ml of heated acetone. This mixture was centrifuged at 5000 –10000 rpm for 5 mins. The supernatant was transferred to another tube and the procedure repeated till the residue became colorless. The absorbance of the solution was read at 480nm, 645nm and 663nm against the solvent (acetone) which was the blank.

**Statistical analysis**

**MALDI-TOF MS**

To perform analyses on the MALDI-TOF MS spectra and the final character data set, various similarity measures were evaluated. Band peaks were used to calculate similarities with available data sets to produce comparable identification results.

**Sequence alignment and analysis**

*De novo* assembly of the respective 31 generated raw reads was done using Geneious Prime^®^ version 2022.2.2. Generated consensus sequences were blasted using Basic Local Alignment Search Tool (BLAST) against GenBank database for comparison and identification. Accessions from GenBank with high similarity index were retrieved and alignment done using ClustalW algorithm within Mega version 11.0.13 software. Evolutionary relationship between isolated and GenBank retrieved sequences was computed in Mega version 11.0.13 software .

**Crude Protein method**

Calculations were done using the formulae;

Nitrogen % = (V1– V2) ×N ×F ×0.014×100/V ×100/S

Where; V1=Titre for the sample (ml);

V2-titre for the blank

N=Normality of standard HCL solution

F=Factor of standard HCL solution

V=Volume of diluted digest taken for distillation (10ml)

S=Weight of sample taken (g)

Protein % = Nitrogen × protein factor

**Determination of the free radical scavenging activity**

The radical scavenging activity was calculated using the following formula:

% inhibition of DPPH = {(A_B_ – A_A_)/A_B_} x 100

Where; A_B_ is the absorption of blank sample

A_A_ is the absorption of tested extract solution.

The results were expressed as percentage inhibition of DPPH and mean inhibitory concentrations (IC_50_) determined from a plot of % inhibition of DPPH versus concentration of extract.

**Fatty acid Method**

Crude fat will be calculated as follows**:**

Where; Weight of empty flask (g) = W1,

Weight of flask and extracted fat (g) = W2,

Weight of sample = S

**Estimation of chlorophyll content**

Concentrations of chlorophyll a, chlorophyll b, total chlorophyll and carotenoid were calculated using the following equations:

Total Chlorophyll: 20.2 (A645) + 8.02 (A663)

Chlorophyll a:12.7(A663) – 2.69 (A645) Chlorophyll b: 22.9(A645) – 4.68(A663)

Carotenoid: [A480 + (0.114 (A663) - (0.638 - A645)] × V/1000 × W

**Results**

**Bacterial isolates detected by MALDI-TOF MS from irrigated and non-irrigated larval sources**

Nutrient agar showed growth for both irrigated and non-irrigated larval positive and negative water samples. A total of seven bacterial genera were identified using MALDI-TOF MS namely *Bacillus, Brevibacillus, Citrobacter, Enterobacter, Escherichia, Klebsiella, and Shigella*. From larval source in non-irrigated zone, only *Bacillus* (*Bacillus amyloliquefaciens*) was identified whereas the rest of genera were from larval sources in irrigated zones. *Shigella* was the dominant (24/51, 47%) genera among the isolates followed by *Escherichia* (13/51, 25%) whereas *Bacillus* (4/51, 8%), *Citrobacter* (4/51, 8%), *Brevibacillus* (2/51, 4%), *Enterobacter* (2/51, 4%), and *Klebsiella* (2/51, 4%) were observed at a lower frequency (**Table 2**). *Escherichia coli* was the abundant species (13/51) followed by *Shigella sonnei* (11/51), *Shigella boydii* (8/51), *Shigella flexneri* (3/51), and *Bacillus amyloliquefaciens* (3/51). The rest *Citrobacter freundi*, *Citrobacter braaki*, and *Klebsiella pneumoniae* were each detected twice (**Fig. 1**). Other bacteria species identified included *Enterobacter cloacae*, *Shigella sp.*, *Enterobacter asburiae*, *Shigella dysentriae*, *Bacillus subtilis*, *Brevibacillus agri*, and *Brevibacillus brevi* (**Fig. 1**).

**Table 2. Distribution of bacterial genera detected by MALDI-TOF MS from irrigated and non-irrigated larval sources.**

| **Genera** | **Larval positive habitats in the irrigated area (n)** | **Larval negative habitats in the irrigated area (n)** | **Larval positive habitats in the non- irrigated area (n)** | **Larval negative habitats in the non- irrigated area (n)** |
| --- | --- | --- | --- | --- |
| ***Bacillus*** | 1 | 2 | - | 1 |
| ***Brevibacillus*** | 2 | 0 | - | - |
| ***Citrobacter*** | 0 | 4 | - | - |
| ***Enterobacter*** | 2 | 0 | - | - |
| ***Escherichia*** | 7 | 6 | - | - |
| ***Klebsiella*** | 1 | 1 | - | - |
| ***Shigella*** | 10 | 14 | - | - |

n: Number of isolates

**Figure 1.** Distribution of bacterial species detected by MALDI-TOF MS from irrigated and non-irrigated larval sources.

Key gram-positive bacteria species in larval present habitats in irrigated areas included *B. amyloliquefaciens*, *B. agri*, and *B. brevi* whereas gram negative included *E. asburiae*, *E. cloacae*, *K. pneumoniae*, *S. boydii*, *S. dysentriae*, *S. flexneri*, and *S. sonnei*. In larval negative habitats gram positive bacteria included *B. amyloliquefaciens*, and *B. subtilis*, whereas gram negative bacteria were *C. braaki*, *C. freundi*, *E. coli*, *K. pneumoniae*, *S. boydii*, *S. flexneri*, *S. sonnei*, and *Shigella* spp. Larval sources in irrigated areas had high bacterial abundance and diversity (**Fig.2**). Habitats within irrigated area that lacked larvae had higher bacterial species abundance as compared to habitats with larvae (27 versus 23). Both habitat types however had nearly equal bacterial species diversity (11 versus 10 in irrigated larval positive sources and those without respectively). From non-irrigated area, only one bacteria species was detected in habitats that lacked larvae (**Fig. 2**).

**Figure 2.** Bacterial species diversity and abundance within aquatic habitats with larval presence or absence within irrigated and non-irrigated areas as detected by MALDI-TOF MS.

**Bacterial isolates identified through sequencing irrigated and non-irrigated larval sources**

Of the 31 sequenced isolates, 65% (20/31) were *Bacillus*, 13% (4/31) were *Escherichia*, 10% (3/31) were *Exiguobacterium*, and 3% (1/31) each were *Paenibacillus*, *Staphylococcus*, *Citrobacter*, and *Enterococcus* (**Table 3**). Among *Bacillus*, *B. velezensis* were the abundant species at a frequency of 25% (5/20), followed by *B. subtilis* 20% (4/20), *B. siralis*, *B. stercoris*, *B. aerius* and *B. cereus* each at 10% (2/20). The rest (*B. inaquosorum*, *B. mojavensis*, and *B. tequilensis*) were at a frequency of 5% (1/20) each **(Table 3)**. The isolates clustered into 12 clades which were representative of the mentioned genera (**Fig.3**). *B. subtilis* (KC441741), *B. mojavensis* (OP218479 and OP482166), *B. tequilensis* (OP435764), and *B. stercoris* (OP521932) clustered with isolate NA10A, NA10B, NA12AA, NA4BB and NA5AA in clade 1. *B. aerius*(OP115492) and isolates EMB6A, EMB6 were in clade 2. Clade 3 comprised of *Robertmurraya siralis* (OK570087), *B. siralis* (MH746088), and isolate NA4A; Clade 4 had *Staphylococcus arlettae* (OP402856) and isolate NA4A; Clade 5 had *E. gallinarum* (OP501807) and isolate NA9; Clade 6 had *B. cereus* (ON860698 and ON430535) and isolate NA3 and MC2; Clade 7 had *Exiguobacterium profundum*(OP793848 and OP263689) and isolates MC1A and NA8AA; Clade 8 had *E. coli* (CP102379), Uncultured bacterium (JQ265468) and isolate EMB9A; Clade 9 had *E. coli* (OP514801) and isolates NA8A, NA11A, MC5B, EMB9 and NASB; Clade 10 had *B. inaquosorium* (ON999044), *B. velezensis* (OP554433), *B. subtilis* (KF732994) isolates NA7C, NA2BA, NA5AB, NA6B, NA4BA, NA12B, and NA12AB; Clade 11 had *Citrobacter sp*. (OX245674) and isolate MC9 while Clade 12 comprised of *B. siralis* (LC588555), *Paenibacillus dendritiformis* (OX216966) and isolates NA2A, and NA7 (**Fig. 3**).

**Table 3. Frequency of isolated Genera** **from larval source in irrigated and non-irrigated areas based on sequencing and blasting results.**

| **Genera** | **Habitats in the irrigated area with**  **larval presence (n)** | **Habitats in the irrigated area with larval absence (n)** | **Habitats in the non-Irrigated area with larval presence (n)** | **Habitats in the non-Irrigated area with larval absence (n)** |
| --- | --- | --- | --- | --- |
| ***Bacillus*** | 6 | 5 | 3 | 6 |
| ***Citrobacter*** | 0 | 1 | 0 | 0 |
| ***Enterococcus*** | 0 | 1 | 0 | 0 |
| ***Escherichia*** | 0 | 4 | 0 | 0 |
| ***Exiguobacterium*** | 3 | 0 | 0 | 0 |
| ***Paenibacillus*** | 0 | 1 | 0 | 0 |
| ***Staphylococcus*** | 0 | 0 | 1 | 0 |

**Figure 3.** Phylogenetic relationship between isolated bacteria and accessions within GenBank.

The evolutionary relationship of the 54 nucleotide sequences, 31 of which were local isolates and 23 were accessions retrieved from GenBank was inferred using Maximum Likelihood method and Tamura-Nei model. The tree with the highest log likelihood (-1502.82) is shown. The percentage of trees in which the associated taxa clustered together is shown next to the branches. Initial tree(s) for the heuristic search were obtained automatically by applying Neighbor-Join and BioNJ algorithms to a matrix of pairwise distances estimated using the Tamura-Nei model, and then selecting the topology with superior log likelihood value. A discrete Gamma distribution was used to model evolutionary rate differences among sites (5 categories (+G, parameter = 6.3041)).

A Pearson's product-moment correlation was computed to assess the relationship between bacterial species abundance in larval sources in irrigated and non-irrigated areas and presence or absence of irrigation. There was a strong, positive correlation between bacterial species abundance in larval sources in irrigated areas and presence of irrigation, which was statistically significant (r=0.334, n=44, p=0.027). Only gram-positive bacteria from the genera *Bacillus* and *Staphylococcus* were found in larval sources in non-irrigated area, with or without larvae. *Staphylococcus arlettae*, *Bacillus cereus*, *B. mojavensis*, *B. siralis*, *B. stercoris*, *B. subtilis*, and *B. velezensis* were among the species isolated. Except for *S. arlettae, B. stercoris*, and *B. velezensis* the other *Bacillus* species were found in non-larval-infested habitats. Only gram-positive bacteria, including *B. aerius*, *B. siralis*, *B. subtilis*, *B. tequilensis*, and *Exiguobacterium profundum*, were found in larval sources with larvae in irrigated areas. Potential larval sources with no larvae in irrigated areas had gram negative (*Citrobacter sp*., *E. coli*, and Uncultured bacterium clone T2C182) and gram-positive bacteria (*B. cereus*, *B. inaquosorum*, *B. stercoris*, *B. subtilis*, *B. velezensis*, *P.dendritiformis*, and *E. gallinarum*).

Generally, there was high bacterial abundance in each area as compared to diversity (**Fig. 4**). Larval positive habitats in irrigated and non-irrigated areas exhibited low bacterial diversity (7 and 2 respectively) as compared to larval negative habitats in the same area (10 and 3, respectively). In terms of abundance, the habitats with larvae in irrigated and non-irrigated areas displayed varied pattern. The habitats in irrigated area had low abundance compared to habitats without larvae within the same area (9 versus 12, respectively). However, in the non-irrigated area, aquatic habitats with larvae had high abundance than habitats with no larvae (6 versus 4, respectively). Habitats with no larvae in irrigated areas had highest bacterial abundance and diversity as compared to the rest followed closely by habitats within the same area with larvae (**Fig. 4**).

**Figure 4.** Bacterial species diversity and abundance within habitats with larval presence or absence within irrigated and non-irrigated areas detected via sequencing.

**Metabolite analysis**

Measurements were gone for water samples with and without mosquito larvae from the irrigated and non- irrigated areas. These were each done in triplicate.

**Fatty acids concentration and larval availability**

There was varying concentration (0.95%-28.42%) of fatty acids (Palmitic, Palmitoleic, Stearic, Oleic, Linoleic, Linolenic, Arachidonic, Eicosapentaenoic acid (Epa), Docosahexaenoic acid (Dha), and Nervonic) in larval habitats within irrigated and non-irrigated areas with larval presence or absence (**Table 4**). Fatty acid concentrations in habitats with larvae within irrigated and non-irrigated areas, and habitats with no larvae in irrigated and non-irrigated areas did not differ significantly, implying that they had no overall effect on larval presence or absence (*F* (3, 36) = 6.732e-008, P>0.99). At the fatty acid component level, larval presence was observed in irrigated and non-irrigated areas where Stearic concentrations were low (5.6% and 6.3% in irrigated and non-irrigated areas, respectively). Contrary to what was observed at low Stearic concentrations, larval presence was observed at elevated Nervonic concentrations of 6% and 6.2% in irrigated and non-irrigated areas, respectively (**Fig. 5**).

**Table 4. Fatty acid concentrations in larval habitats with and with no larvae within irrigated and non-irrigated areas.**

| **Fatty acids** | **Fatty acid concentration (%) in larval positive habitats in the irrigated area** | **Fatty acid concentration (%) in larval negative habitats in the irrigated area** | **Fatty acid concentration (%) in larval positive habitats in the non- irrigated area** | **Fatty acid concentration (%) in larval negative habitats in the non- irrigated area** |
| --- | --- | --- | --- | --- |
| Palmitic | 28.42 | 28.28 | 13.77 | 22.45 |
| Palmitoleic | 16.49 | 8.74 | 12.39 | 12.36 |
| Stearic | 5.60 | 10.76 | 6.29 | 14.63 |
| Oleic | 14.68 | 11.90 | 12.28 | 26.07 |
| Linoleic | 7.07 | 4.10 | 9.31 | 5.33 |
| Linolenic | 8.31 | 3.40 | 6.86 | 7.28 |
| Arachidonic | 5.99 | 10.45 | 10.01 | 4.92 |
| Epa | 3.98 | 5.27 | 10.65 | 2.19 |
| Dha | 3.44 | 13.38 | 12.22 | 3.82 |
| Nervonic | 6.01 | 3.72 | 6.21 | 0.95 |
| **Average fatty acid** | 10 | 10 | 10 | 10 |

**Figure 5.** Component of fatty acid concentration in habitats with larval presence or absence within irrigated and non-irrigated areas. The concentration of each fatty acid component in larval habitats in an irrigated area (**A**). The concentration of each fatty acid component in larval habitats in non-irrigated area (**B**).

**Phytochemical concentration and larval availability**

In both larval breeding sites in irrigated and non-irrigated areas, the crude fat content ranged from 0.01% to 0.18%. Larval positive habitats in the irrigated area had a lower crude fat content of 0.01% than the same site without larvae (0.18%). The crude fat content (0.01%) was the same in non-irrigated area with and without larvae. Larval habitats within irrigated areas also recorded high crude protein content (0.13% and 0.16% in larval habitats within irrigated areas with and with no larvae, respectively) compared to those in non-irrigated areas (0.08% and 0.07%). Larvae were found in irrigated areas with low crude protein content (0.13% versus 0.16%) as opposed to what was observed in non-irrigated areas (0.08% versus 0.07%). Similar observation was made on Beta-Carotene(µg/100g) content in irrigated areas (1.26 versus 2.04) and non-irrigated areas (5.2 versus 3.16) as well as total phenol µg (GAE)/100g (**Table 5)**. Larval presence was evident in aquatic habitats within irrigated and non-irrigated areas with high total chlorophyll content (1.12 µg/g versus 0.81µg/g and 3.37 µg/g versus 0.82). Both Tannin and anti-oxidant contents were low in habitats with larvae in irrigated areas and high in habitats with larvae in non-irrigated areas (**Table 5**). The varied concentration of each phytochemical component between habitats with larvae and with no larvae in irrigated and non-irrigated areas was not significant.

**Table 5. Phytochemical concentrations in larval habitats with and with no larvae within irrigated and non-irrigated areas.**

| **Element** | **Larval positive habitats in the irrigated area** | **Larval negative habitats in the irrigated area** | **Larval positive habitats in the non-irrigated area** | **Larval negative habitats in the non- irrigated area** |
| --- | --- | --- | --- | --- |
| Crude Fat (%) | 0.04 | 0.18 | 0.01 | 0.01 |
| Crude Protein (%) | 0.13 | 0.16 | 0.08 | 0.07 |
| Beta-Carotene (µg/100g) | 1.26 | 2.04 | 5.20 | 3.16 |
| Total Phenol (µg (GAE)/100g) | 4.49 | 5.29 | 4.55 | 4.68 |
| Total Chlorophyll (µg/g) | 1.12 | 0.81 | 3.37 | 0.82 |
| Tannin (mg (TAE)/100g) | 0.10 | 1.08 | 3.22 | 0.28 |
| Anti-oxidant (@0.5ml/ml) % Inhibition | 20.38 | 29.40 | 26.40 | 23.65 |

**Sugar concentration and larval availability**

Habitats with larvae in irrigated areas had high concentration (mg/100ml) of Pectin (0.64 versus 0.50), cellulose (7.72 versus 2.86), and fructose (0.36 versus 0.33) as compared habiats with no larvae. Similar trend on pectin, cellulose and fructose was observed in habitats with and with no larvae in irrigated areas (**Table 6**). The three sugar components were at higher concentration in larval habitats within non-irrigated areas with larvae as compared to habitats with larvae in irrigated areas (**Fig. 6**). Glucose concentration was however low in habitats with larvae in irrigated areas compared to habitats with no larvae (0.38 versus 1.16). In non-irrigated areas, glucose concentration was high in habitats with larvae as compared to those without (2.67 versus 0.47). Similar observation was made on sucrose concentration in irrigated (0.08 versus 0.16) and non-irrigated areas (0.25 versus 0.09). The varied concentration of each sugar component between habitats with larvae and with no larvae in irrigated and non-irrigated areas was not significant.

**Table 6. Sugar concentrations in larval habitats with and with no larvae within irrigated and non-irrigated areas.**

| Components | **Larval positive habitats in the irrigated area** | **Larval negative habitats in the irrigated area** | **Larval positive habitats in the non- irrigated area** | **Larval negative habitats in the non- irrigated area** |
| --- | --- | --- | --- | --- |
| Pectin(mg/100ml) | 0.64 | 0.50 | 0.77 | 0.19 |
| Cellulose(mg/100ml) | 7.72 | 2.86 | 11.17 | 0.86 |
| Glucose(mg/100ml) | 0.38 | 1.16 | 2.67 | 0.47 |
| Fructose(mg/100ml) | 0.36 | 0.33 | 0.76 | 0.25 |
| Sucrose(mg/100ml) | 0.08 | 0.16 | 0.25 | 0.09 |

**Figure 6.** Component of sugar concentration in habitats with larval presence or absence within irrigated and non-irrigated areas. The concentration of each sugar component in larval habitats in an irrigated area (**A**). The concentration of each sugar component in larval habitats in non-irrigated area (**B**).

**Mineral concentration and larval availability**

High concentration of Manganese, Calcium and Copper were observed in habitats with larvae in irrigated and non-irrigated areas as compared to those without (**Table 7**). Other minerals such as Lead, Iron, Magnesium and Zinc were at near similar concentration in all habitats (**Fig. 7**).

**Table 7.** Mineral concentrations in larval habitats with and with no larvae within irrigated and non-irrigated areas.

| **Minerals** | **Larval positive habitats in the irrigated area** | **Larval negative habitats in the irrigated area** | **Larval positive habitats in the non- irrigated area** | **Larval negative habitats in the non- irrigated area** |
| --- | --- | --- | --- | --- |
| Lead (mg/100ml) | 5.70 | 5.41 | 8.62 | 9.47 |
| Manganese (mg/100ml) | 249.72 | 153.83 | 198.04 | 182.48 |
| Calcium (mg/100ml) | 8.46 | 7.89 | 9.10 | 6.51 |
| Copper (mg/100ml) | 4.97 | 3.66 | 6.25 | 5.00 |
| Iron (mg/100ml) | 1.16 | 1.08 | 1.11 | 1.16 |
| Magnesium (mg/100ml) | 1.12 | 1.12 | 1.16 | 1.12 |
| Zinc (mg/100ml) | 0.02 | 0.00 | 0.01 | 0.01 |

**Figure 7.** Mineral concentration in habitats with larval presence or absence within irrigated and non-irrigated areas. The concentration of minerals in larval habitats in an irrigated area (**A**). The concentration of minerals in larval habitats in non-irrigated area (**B**).

**Discussion**

This study revealed important aspects that can be used in the development of vector control tools. *Shigella* and *Bacillus* genera were observed to be dominant in habitats in the irrigated and non- irrigated areas respectively. Irrigation positively influenced bacterial abundance and diversity. Larvicidal isolates *Brevibacillus brevis*, *Bacillus subtilis*, and *Exiguobacterium profundum* were isolated and grouped with *Bacillus mojavensis*, *Bacillus tequilensis*, *Bacillus stercoris*, and *Brevibacillus agri*. Less proteins and fats were detected in the irrigated area from both larval positive and larval negative habitats as compared to the non- irrigated area, while high chlorophyll and sugar content was associated with larval presence in both areas. In addition, substantial concentrations of Manganese, Calcium, and Copper were found in sites containing larvae in both irrigated and non-irrigated areas.

*Bacillus amyloliquefaciens*, a subspecies of the *B. subtilis* is known to be bio-control against plant pathogens . This species has also been observed to reduce harmful water components that reduce water oxygen content like ammonia, total nitrogen, total phosphorus and chemical oxygen demand while increasing nitrates . The presence of this species as the only bacteria identified via MALDI-TOF in the non- irrigated area confirms that *B. amyloliquefaciens* signiﬁcantly affects the [bacterial community](https://www.sciencedirect.com/topics/agricultural-and-biological-sciences/bacterial-communities) diversity and composition in the non- irrigated area. This is similar to studies done by Yang and others . The presence of *B. amyloliquefaciens* in the long standing aquatic habitats in the non- irrigated area confirms that the species does not colonize a native bacterial community in a freshwater aquatic environment .

Based on MALDI-TOF MS and sequencing results, larval sources in irrigated areas had higher bacterial species diversity and abundance than sources in non-irrigated areas. Similarly to previous research that linked increased soil bacterial abundance and diversity to irrigation , the findings of this study confirm the influence of irrigation on increased bacterial abundance and species diversity that may influence larval productivity on larval sources or pools. MALDI-TOF MS, revealed *Shigella* and *Escherichia* as the dominant genera in larval sources with larvae presence in irrigated areas. Except for *Brevibacillus*, some species of the former two and other isolated genera (*Bacillus*, *Klebsiella*, *Citrobacter*, and *Enterobacter*) are considered endosymbiont and have been isolated from the gut of *Anopheles* . With the exception of *Bacillus sphaericus*, which reduces vectoral capacity , species from the five genera have a significant influence on nutrient assimilation, vectoral capacity, and membrane formation in mosquitoes, explaining the importance of their presence in Homa Bay larval sources . Members of the genera have also been linked to the synthesis of kairomones that attract and stimulate oviposition in vectors such as *Aedes aegypti* and *Ae. Albopictus* .

The exempted genus *Brevibacillus* has previously been linked to entomopathogenic activities, with the spores produced by *Brevibacillus laterosporus* and *Brevibacillus brevis* being highly toxic to both larvae and adult mosquitoes . The presence of *Brevibacillus brevis* may have an effect on larval densities in larval sources with larvae in irrigated areas in the study site, interfering with their overall larval productivity. Furthermore, because *Brevibacillus agri* is closely related to *Brevibacillus brevis*, it should be tested for entomopathogenic characteristics . This will determine whether the bacteria play a role in larval reduction or forms bio-larvicidal spores and can be used in larval source management (LSM).

Additional four bacterial taxa (*Enterococcus*, *Exiguobacterium*, *Paenibacillus*, and *Staphylococcus*) were found in larval sources in irrigated and non-irrigated parts of Homa Bay, according to sequencing data. Among the key identified bacteria that were only present in larval present sources in irrigated areas of Homa Bay was *Exiguobacterium profundum*. The bacterium is known to impair mosquito fecundity, egg hatchability, and larval development, and it may have a detrimental impact on larval productivity in larval sources inside irrigated regions . Although present in potential larval habitats that lacked *Anopheles* larvae, *Bacillus subtilis* is renowned for generating bio-surfactant surfactin, which is a bio-adulticide to *An. stephensi* , as well as other secondary metabolites that are bio-larvicides to *A. aegypti* . The finding of clustering or grouping with other isolated bacteria such as *B. mojavensis*, *B. tequilensis*, and *B. stercoris* (found in larvae infested larval sources) suggests that the larvicidal or adulticidal capability of these bacteria should be investigated.

Stearic and oleic acids are fatty acids with long hydro-carbon chains. Absence of larvae in aquatic habitats with high levels of stearic acid could be as a result of the nature of this fatty acid. Stearic acid is known to be waxy and slightly soluble in water thus forming a thin layer on water surfaces . This layer restricts entry of oxygen into the water. This could be a hindrance to oviposition adult mosquitoes as they tend to prefer habitats that will support the survival of the immature. Stearic acid has been observed to be potent to mosquito larvae . Oleic acid, a monounsaturated fatty acid, soluble in water, is known to be beneficial to other living organisms. However oleic and linoleic acids have been observed to be lethal to mosquito larvae in high concentrations . This could be the reason for the optimal concentrations of these fatty acids in habitats with larvae. Linoleic acid has been observed to be beneficial for mosquito adult development .

A high concentration of total chlorophyll was observed to support habitat infestation by mosquito larvae. This could be due to the presence of algae matter which acts as food for the immature to support their growth. This is consistent with other studies that observed that algae was used as food for mosquito larvae .

The nutritional capacity of mosquito larvae is essential for the development of larvae and emerging adult mosquitoes. Larvae with inadequate nutrition may result in developmental failure or have a negative effect on the size and reproductive ability of resulting adult mosquitoes. Generally, high concentrations of simple sugar were associated with larval presence in the habitats. This was observed for pectin, cellulose, glucose, fructose and sucrose. These are known as soluble and ready food products that are used by mosquito larvae for survival.

Similarly, manganese was found at higher concentrations than other metals throughout larval habitats in irrigated and non-irrigated regions with larval presence. Manganese, in conjunction with calcium, chlorine, iron, potassium, magnesium, sodium, sulfur, and phosphorus, is known to promote normal mosquito larval development . The majority of the other metals, including lead, calcium, copper, iron, magnesium, and zinc, were found in extremely low concentrations. Although copper is required for enzyme functioning in larval pigmentation, oxidative stress protection, and respiration, excessive concentrations are known to be larvicidal , and has also been linked to mosquito resistance to insecticides such as lambda-cyhalothrin .

**Conclusion**

Irrigation increased bacterial species abundance and diversity in larval sources within the irrigated area**.** *Shigella* and *Escherichia* species were the common genera in larval sources with larvae presence in irrigated areas. In the irrigated region, notable larvicidal bacteria *B. brevis*, *B. subtilis,* and *E. profundum* were found. Moreover, the larvicidal or adulticidal properties of *B. mojavensis*, *B. tequilensis*, *B. stercoris*, and *Brevibacillus agri* should be examined since they clustered with known entomopathogenic bacteria in this study. In addition, high fatty acid concentration may have an impact on larval production in both irrigated and non-irrigated areas, whereas chlorophyll, sugar, and manganese promoted growth.

**Conflict of interest:**

All authors declare that they have no conflict of interest.

**Authors’ contributions:**

PWO, AKG and SNG conceived and designed the research.PWO, KO, GY and HA participated in the design and implementation of entomological studies. PWO and KO did the data analysis. PWO and KO drafted the first manuscript. AKG, HA, GY and SGN edited the manuscript. All authors read and approved the final version of the manuscript.

**Acknowledgments:**

The authors wish to thank the community and the leaders of Homa Bay County for allowing us to work in the area. We also acknowledge the sub-Saharan Africa ICEMR field team and staff for providing professional and technical support during the study; more specifically Sally Mongoi, Fredrick Odongo and Thomas Katiye. In addition, we would like to thank Anne Owiti, Sebastian Musundi and David Abuga for their technical support provided.

**Funding**

The work was supported by grants from the International Foundation for Science (I2-A-6605-1), Mawazo Research Funds (2022-0-07 and 2022-3-09)

**References**

1.	Coetzee M, Craig M, Le Sueur D. Distribution of African malaria mosquitoes belonging to the Anopheles gambiae complex. Parasitology today. 2000;16(2):74-7.

2.	Manguin S, Garros C, Dusfour I, Harbach R, Coosemans M. Bionomics, taxonomy, and distribution of the major malaria vector taxa of Anopheles subgenus Cellia in Southeast Asia: an updated review. Infection, Genetics and Evolution. 2008;8(4):489-503.

3.	Sinka ME, Bangs MJ, Manguin S, Chareonviriyaphap T, Patil AP, Temperley WH, et al. The dominant Anopheles vectors of human malaria in the Asia-Pacific region: occurrence data, distribution maps and bionomic précis. Parasites & vectors. 2011;4(1):1-46.

4.	Sinka ME, Bangs MJ, Manguin S, Rubio-Palis Y, Chareonviriyaphap T, Coetzee M, et al. A global map of dominant malaria vectors. Parasites & vectors. 2012;5(1):1-11.

5.	Roberts DR, Manguin S, Rejmankova E, Andre R, Harbach RE, Vanzie E, et al. Spatial distribution of adult Anopheles darlingi and Anopheles albimanus in relation to riparian habitats in Belize, Central America. Journal of Vector Ecology. 2002;27:21-30.

6.	Minakawa N, Sonye G, Mogi M, Githeko A, Yan G. The effects of climatic factors on the distribution and abundance of malaria vectors in Kenya. Journal of medical entomology. 2002;39(6):833-41.

7.	Okara RM, Sinka ME, Minakawa N, Mbogo CM, Hay SI, Snow RW. Distribution of the main malaria vectors in Kenya. Malaria journal. 2010;9(1):1-11.

8.	Mwingira V, Mboera LE, Dicke M, Takken W. Exploiting the chemical ecology of mosquito oviposition behavior in mosquito surveillance and control: a review. Journal of Vector Ecology. 2020;45(2):155-79.

9.	Gimnig JE, Ombok M, Kamau L, Hawley WA. Characteristics of larval anopheline (Diptera: Culicidae) habitats in Western Kenya. Journal of medical entomology. 2001;38(2):282-8.

10.	Spencer M, Blaustein L, Cohen JE. Oviposition habitat selection by mosquitoes (Culiseta longiareolata) and consequences for population size. Ecology. 2002;83(3):669-79.

11.	Minakawa N, Sonye G, Mogi M, Yan G. Habitat characteristics of Anopheles gambiae ss larvae in a Kenyan highland. Medical and veterinary entomology. 2004;18(3):301-5.

12.	Okal MN, Francis B, Herrera-Varela M, Fillinger U, Lindsay SW. Water vapour is a pre-oviposition attractant for the malaria vector Anopheles gambiae sensu stricto. Malaria journal. 2013;12(1):1-8.

13.	Himeidan YE, Temu EA, El Rayah EA, Munga S, Kweka EJ. Chemical cues for malaria vectors oviposition site selection: challenges and opportunities. Journal of Insects. 2013;2013:1-9.

14.	Rejmánková E, Higashi R, Grieco J, Achee N, Roberts D. Volatile substances from larval habitats mediate species-specific oviposition in Anopheles mosquitoes. Journal of Medical Entomology. 2005;42(2):95-103.

15.	Sumba LA, Guda TO, Deng AL, Hassanali A, Beier JC, Knols BG. Mediation of oviposition site selection in the African malaria mosquito Anopheles gambiae (Diptera: Culicidae) by semiochemicals of microbial origin. International Journal of Tropical Insect Science. 2004;24(3):260-5.

16.	Lindh JM, Okal MN, Herrera-Varela M, Borg-Karlson A-K, Torto B, Lindsay SW, et al. Discovery of an oviposition attractant for gravid malaria vectors of the Anopheles gambiae species complex. Malaria Journal. 2015;14(1):1-12.

17.	Girard M, Martin E, Vallon L, Raquin V, Bellet C, Rozier Y, et al. Microorganisms associated with mosquito oviposition sites: Implications for habitat selection and insect life histories. Microorganisms. 2021;9(8):1589.

18.	Mulamba C, Riveron JM, Ibrahim SS, Irving H, Barnes KG, Mukwaya LG, et al. Widespread pyrethroid and DDT resistance in the major malaria vector Anopheles funestus in East Africa is driven by metabolic resistance mechanisms. PLoS One. 2014;9(10):e110058.

19.	Arbaoui A, Chua T. Bacteria as a source of oviposition attractant for Aedes aegypti mosquitoes. Trop Biomed. 2014;31(1):134-42.

20.	Mokany A, Shine R. Oviposition site selection by mosquitoes is affected by cues from conspecific larvae and anuran tadpoles. Austral Ecology. 2003;28(1):33-7.

21.	Kramer WL, Mulla MS. Oviposition attractants and repellents of mosquitoes: oviposition responses of Culex mosquitoes to organic infusions. Environmental Entomology. 1979;8(6):1111-7.

22.	Takken W, Knols BG. Odor-mediated behavior of Afrotropical malaria mosquitoes. Annual review of entomology. 1999;44(1):131-57.

23.	Otienoburu P, Bayoh N, Gimnig J, Huang J, Walker E, Otieno M, et al. Anopheles gambiae (Diptera: Culicidae) oviposition as influenced by type of water infused into black and red soils of western Kenya. International Journal of Tropical Insect Science. 2007;27:2-5.

24.	Sumba LA, Okoth K, Deng AL, Githure J, Knols BG, Beier JC, et al. Daily oviposition patterns of the African malaria mosquito Anopheles gambiae Giles (Diptera: Culicidae) on different types of aqueous substrates. Journal of circadian rhythms. 2004;2(1):1-7.

25.	Benzon GL, Apperson CS. Reexamination of chemically mediated oviposition behavior in Aedes aegypti (L.)(Diptera: Culicidae). Journal of Medical Entomology. 1988;25(3):158-64.

26.	Rodriguez M. Limnological and botanical characterization of larval habitats for two primary malarial vectors, *Anopheles* A lbimanus and Anopheles pseudopunctipennis in coastal areas of Chiapas State, Mexico. 1990.

27.	Himeidan YE, Zhou G, Yakob L, Afrane Y, Munga S, Atieli H, et al. Habitat stability and occurrences of malaria vector larvae in western Kenya highlands. Malar J. 2009;8:234.

28.	Kibret S, Wilson GG, Ryder D, Tekie H, Petros B. Malaria impact of large dams at different eco-epidemiological settings in Ethiopia. Tropical medicine and health. 2017;45(1):1-14.

29.	Debrah I, Afrane YA, Amoah LE, Ochwedo KO, Mukabana WR, Zhong D, et al. Larval ecology and bionomics of Anopheles funestus in highland and lowland sites in western Kenya. PloS one. 2021;16(10):e0255321.

30.	Orondo PW, Wang X, Lee M-C, Nyanjom SG, Atieli H, Ondeto BM, et al. Habitat Diversity, Stability, and Productivity of Malaria Vectors in Irrigated and Nonirrigated Ecosystems in Western Kenya. Journal of Medical Entomology. 2022.

31.	Torres-Estrada JL, Meza-Alvarez RA, Cibrián-Tovar J, Rodríguez-Lopez MH, Arredondo-Jiménez JI, Cruz-López L, et al. Vegetation-derived cues for the selection of oviposition substrates by Anopheles albimanus under laboratory conditions. Journal of the American Mosquito Control Association. 2005;21(4):344-9.

32.	Pates H, Curtis C. Mosquito behavior and vector control. Annu Rev Entomol. 2005;50:53-70.

33.	Ondeto BM, Wang X, Atieli H, Orondo PW, Ochwedo KO, Omondi CJ, et al. Malaria vector bionomics and transmission in irrigated and non-irrigated sites in western Kenya. Parasitology Research. 2022:1-17.

34.	Omondi CJ, Wilfred OO, Odongo D, Ochwedo KO, Otieno A, Onyango SA, et al. Asymptomatic and Submicroscopic Plasmodium Infections in an Area Before and During Integrated Vector Control in Homa Bay, Western Kenya. 2022.

35.	Feldsine P, Abeyta C, Andrews WH. AOAC International methods committee guidelines for validation of qualitative and quantitative food microbiological official methods of analysis. Journal of AOAC international. 2002;85(5):1187-200.

36.	Waterman PG, Mole S. Analysis of phenolic plant metabolites: Blackwell Scientific; 1994.

37.	Bligh EG, Dyer WJ. A rapid method of total lipid extraction and purification. Canadian journal of biochemistry and physiology. 1959;37(8):911-7.

38.	Molyneux P. The use of the stable radical Diphenylpicrylhydrazyl (DPPH) for estimating antioxidant activity, 26. 2003.

39.	Williams S. AOAC official methods of analysis. Association of Official Analytical Chemists, 14th Edition, Arlington, VA. 1984:8-34.

40.	Arnon DI. Copper enzymes in isolated chloroplasts. Polyphenoloxidase in Beta vulgaris. Plant physiology. 1949;24(1):1.

41.	Tamura K, Stecher G, Kumar S. MEGA11: molecular evolutionary genetics analysis version 11. Molecular biology and evolution. 2021;38(7):3022-7.

42.	Tran TD, Del Cid C, Hnasko R, Gorski L, McGarvey JA. Bacillus amyloliquefaciens ALB65 inhibits the growth of Listeria monocytogenes on cantaloupe melons. Applied and environmental microbiology. 2020;87(1):e01926-20.

43.	El-Hefny A, Khalil OA, Hassan YE, Mounir AM. Biocontrol effect of Bacillus amyloliquefaciens combined with un-irradiated and irradiated beet root as a preservative coating for pomegranate arils. Journal of Radiation Research and Applied Sciences. 2022;15(2):33-45.

44.	Asari S, Matzén S, Petersen MA, Bejai S, Meijer J. Multiple effects of Bacillus amyloliquefaciens volatile compounds: plant growth promotion and growth inhibition of phytopathogens. FEMS Microbiology Ecology. 2016;92(6):fiw070.

45.	Yang Y, Xia J, Liu Y, Dong J, Xu N, Yang Q, et al. Safety evaluation for the use of Bacillus amyloliquefaciens in freshwater fish cultures. Aquaculture Reports. 2021;21:100822.

46.	Zhou T, Zheng X, Tang J, Qin J, Wang Y. Effect of three commercial microbial products on bacterial community in a freshwater fish polyculture system. Aquaculture Research. 2017;48(8):4449-60.

47.	Li H, Wang H, Jia B, Li D, Fang Q, Li R. Irrigation has a higher impact on soil bacterial abundance, diversity and composition than nitrogen fertilization. Scientific Reports. 2021;11(1):16901.

48.	Frene JP, Faggioli V, Covelli J, Reyna D, Gabbarini LA, Sobrero P, et al. Agriculture by irrigation modifies microbial communities and soil functions associated with enhancing C uptake of a steppe semi-arid soil in northern Patagonia. Frontiers in Soil Science. 2022:7.

49.	Gendrin M, Christophides GK. The Anopheles mosquito microbiota and their impact on pathogen transmission. Anopheles mosquitoes-New insights into malaria vectors: IntechOpen; 2013.

50.	Galeano-Castañeda Y, Urrea-Aguirre P, Piedrahita S, Bascuñán P, Correa MM. Composition and structure of the culturable gut bacterial communities in Anopheles albimanus from Colombia. Plos one. 2019;14(12):e0225833.

51.	Chen S, Zhang D, Augustinos A, Doudoumis V, Bel Mokhtar N, Maiga H, et al. Multiple factors determine the structure of bacterial communities associated with Aedes albopictus under artificial rearing conditions. Frontiers in Microbiology. 2020;11:605.

52.	Yu S, Wang P, Qin J, Zheng H, Wang J, Liu T, et al. Bacillus sphaericus exposure reduced vector competence of Anopheles dirus to Plasmodium yoelii by upregulating the Imd signaling pathway. Parasites & Vectors. 2020;13:1-10.

53.	Minard G, Mavingui P, Moro CV. Diversity and function of bacterial microbiota in the mosquito holobiont. Parasites & vectors. 2013;6(1):1-12.

54.	Coon KL, Vogel KJ, Brown MR, Strand MR. Mosquitoes rely on their gut microbiota for development. Molecular ecology. 2014;23(11):2727-39.

55.	Rodgers FH, Gendrin M, Wyer CA, Christophides GK. Microbiota-induced peritrophic matrix regulates midgut homeostasis and prevents systemic infection of malaria vector mosquitoes. PLoS pathogens. 2017;13(5):e1006391.

56.	Ponnusamy L, Xu N, Nojima S, Wesson DM, Schal C, Apperson CS. Identification of bacteria and bacteria-associated chemical cues that mediate oviposition site preferences by Aedes aegypti. Proceedings of the National Academy of Sciences. 2008;105(27):9262-7.

57.	Barbieri G, Ferrari C, Mamberti S, Gabrieli P, Castelli M, Sassera D, et al. Identification of a Novel Brevibacillus laterosporus Strain With Insecticidal Activity Against Aedes albopictus Larvae. Frontiers in microbiology. 2021;12:624014.

58.	Fouda A, Hassan SE-D, Eid AM, Awad MA, Althumayri K, Badr NF, et al. Endophytic bacterial strain, Brevibacillus brevis-mediated green synthesis of copper oxide nanoparticles, characterization, antifungal, in vitro cytotoxicity, and larvicidal activity. Green Processing and Synthesis. 2022;11(1):931-50.

59.	Shida O, Takagi H, Kadowaki K, Udaka S, Komagata K. Bacillus galactophilus is a later subjective synonym of Bacillus agri. International Journal of Systematic and Evolutionary Microbiology. 1994;44(1):172-3.

60.	Rajagopal G, Ilango S. Exposure of Exiguobacterium spp. to dengue vector, Aedes aegypti reduces growth and reproductive fitness. The Journal of Basic and Applied Zoology. 2021;82(1):1-9.

61.	Geetha I, Paily KP, Manonmani AM. Mosquito adulticidal activity of a biosurfactant produced by Bacillus subtilis subsp. subtilis. Pest management science. 2012;68(11):1447-50.

62.	Revathi K, Chandrasekaran R, Thanigaivel A, Kirubakaran SA, Sathish-Narayanan S, Senthil-Nathan S. Effects of Bacillus subtilis metabolites on larval Aedes aegypti L. Pesticide biochemistry and physiology. 2013;107(3):369-76.

63.	Acid L. Final report on the safety assessment of oleic acid, laurie acid, palmitic acid, myristic acid, and stearic acid. J Am Coll Toxicol. 1987;6:321-401.

64.	Rahuman AA, Venkatesan P, Gopalakrishnan G. Mosquito larvicidal activity of oleic and linoleic acids isolated from Citrullus colocynthis (Linn.) Schrad. Parasitology research. 2008;103:1383-90.

65.	Gurunathan A, Senguttuvan J, Paulsamy S. Evaluation of mosquito repellent activity of isolated oleic acid, eicosyl ester from Thalictrum javanicum. Indian Journal of Pharmaceutical Sciences. 2016;78(1):103.

66.	Dadd R, Kleinjan J. Essential fatty acid for the mosquito Culex pipiens: arachidonic acid. Journal of Insect Physiology. 1979;25(6):495-502.

67.	Walker ED, Olds EJ, Merritt RW. Gut content analysis of mosquito larvae (Diptera: Culicidae) using DAPI stain and epifluorescence microscopy. Journal of Medical Entomology. 1988;25(6):551-4.

68.	Gilmar Sidnei E, Donat Peter H, Peter R, Suellen Carolina S, Stephanie W, editors. New Perspectives for the Control Of Mosquito Larvae Using Chlorophyll Derivates As Photosensitizers. ISEE Conference Abstracts; 2013.

69.	Huzortey AA, Kudom AA, Mensah BA, Sefa-Ntiri B, Anderson B, Akyea A. Water quality assessment in mosquito breeding habitats based on dissolved organic matter and chlorophyll measurements by laser-induced fluorescence spectroscopy. Plos one. 2022;17(7):e0252248.

70.	Abdelkader M, El-Tayeb T. Chlorophyll derivative activated with sunlight for malaria vector control: a small-scale field study. 2021.

71.	Bond J, Ramírez-Osorio A, Marina C, Fernández-Salas I, Liedo P, Dor A, et al. Efficiency of two larval diets for mass-rearing of the mosquito Aedes aegypti. PLoS One. 2017;12(11):e0187420.

72.	Rivera-Pérez C, Clifton ME, Noriega FG. How micronutrients influence the physiology of mosquitoes. Current opinion in insect science. 2017;23:112-7.

73.	Reza M, Ilmiawati C, Matsuoka H. Application of copper-based ovitraps in local houses in West Sumatra, Indonesia: a field test of a simple and affordable larvicide for mosquito control. Tropical Medicine and Health. 2016;44(1):1-5.

74.	Reza M, Ilmiawati C. Laboratory testing of low concentration (< 1 ppm) of copper to prolong mosquito pupation and adult emergence time: An alternative method to delay mosquito life cycle. PLoS One. 2020;15(5):e0226859.

75.	Talom AD, Essoung MA, Gbankoto A, Tchigossou G, Akoton R, Sahabi BBA, et al. A preliminary analysis on the effect of copper on Anopheles coluzzii insecticide resistance in vegetable farms in Benin. Scientific reports. 2020;10(1):6392.

**SUPPLIMENTARY FILE**

**Table S1.** Blast results of samples collected from the field as identified by sequencing

| **Area classification** | **Sample identity** | **Blast results** | **Genus** |
| --- | --- | --- | --- |
| Irrigated | MC9 | *Citrobacter sp*. Marseille-Q6884 | *Citrobacter* |
| Irrigated | NA2A | *Paenibacillus dendritiformis* | *Paenibacillus* |
| Irrigated | EMB9A | Uncultured bacterium clone T2C182 | *Escherichia* |
| Irrigated | NA9 | *Enterococcus gallinarum* strain 10 A | *Enterococcus* |
| Irrigated | NA10 | *Bacillus siralis* strain PA02 | *Bacillus* |
| Irrigated | MC2 | *Bacillus cereus* strain CUMB AR-04 | *Bacillus* |
| Irrigated | NA5AA | *Bacillus stercoris* strain ML-2 | *Bacillus* |
| Irrigated | NA10A | *Bacillus tequilensis* strain A37 | *Bacillus* |
| Irrigated | NA10B | *Bacillus subtilis* strain B59 | *Bacillus* |
| Irrigated | MC5B, NA5B, EMB9 | *Escherichia coli* strain ASBY05 | *Escherichia* |
| Irrigated | MC1A, NA8AA, NA8A | *Exiguobacterium profundum* strain H-1 | *Exiguobacterium* |
| Irrigated | EMB6 | *Bacillus aerius* strain GS26 | *Bacillus* |
| Irrigated | NA2BA | *Bacillus velezensis* strain r22 | *Bacillus* |
| Irrigated | NA5AB, NA6B | *Bacillus subtilis* strain PMM8 | *Bacillus* |
| Irrigated | NA2B | *Bacillus inaquosorum* strain GZCB-3 | *Bacillus* |
| Irrigated | EMB6A | *Bacillus aerius* strain GS26 | *Bacillus* |
| Non-Irrigated | NA7 | *Bacillus siralis* J35TS1 | *Bacillus* |
| Non-Irrigated | NA4A | *Staphylococcus arlettae* strain Dg-E8 | *Staphylococcus* |
| Non-Irrigated | NA3 | *Bacillus cereus* strain CUMB AR-04 | *Bacillus* |
| Non-Irrigated | NA4BB | *Bacillus stercoris* strain ML-2 | *Bacillus* |
| Non-Irrigated | NA12AA | *Bacillus mojavensis* strain M | *Bacillus* |
| Non-Irrigated | NA12B, NA4BA, NA7C, NA11A | *Bacillus velezensis* strain r22 | *Bacillus* |
| Non-Irrigated | NA12AB | *Bacillus subtilis* strain PMM8 | *Bacillus* |
