# Supplementary material for: Effects of bacterial composition and aquatic habitat metabolites on malaria vector larval availability in irrigated and non-irrigated sites of Homa Bay county, western Kenya
Source: PLoS One. 2023 Jun 2;18(6):e0286509. doi: 10.1371/journal.pone.0286509 (PMC10237499; doi:10.1371/journal.pone.0286509)
Supplement: Attachment — Submitted filename: Comment Responses.doc [file pone.0286509.s004.doc]

16^th^ May 2023.

Dear Editor, *Plos One*

Dear Sir/ Madam,

Please find enclosed a re-submission to the *Plos One* *Journal,* a research study entitled “**Effects of bacterial composition and aquatic habitat metabolites on malaria vector larval availability in irrigated and non- irrigated sites of Homa Bay county, western Kenya**”. The manuscript is 32 pages long exclusive of 7 tables and 8 figures. 2 additional supplementary tables are also attached.

We express our cordial gratitude for the valuable and constructive review of our paper. The reviewers’ comments have helped us to improve the overall quality of the paper. We have incorporated all the suggestions/ corrections as proposed. Below are the specific responses to the reviewers’ comments.

Kind Regards,

Pauline Orondo

[paulineorondo@gmail.com](mailto:paulineorondo@gmail.com)

**Response to reviewer comments**

The following are our point-by-point responses to the comments raised by the academic editor and reviewers. We thank the editor and reviewers for their constructive criticism and insights that have helped to improve this paper. The responses and referenced from the “track changes document”

| **Academic Editor** | **Responses to the Editor’s comments** |
| --- | --- |
| Please ensure that your manuscript meets PLOS ONE's style requirements, including those for file naming | Formatting has been done as recommended |
| PLOS requires an ORCID iD for the corresponding author in Editorial Manager on papers submitted after December 6th, 2016. Please ensure that you have an ORCID iD and that it is validated in Editorial Manager. To do this, go to ‘Update my Information’ (in the upper left-hand corner of the main menu), and click on the Fetch/Validate link next to the ORCID field. This will take you to the ORCID site and allow you to create a new iD or authenticate a pre-existing iD in Editorial Manager | My ORCID ID has been provided (0000-0002-8800-5994). |
| Thank you for submitting the above manuscript to PLOS ONE. During our internal evaluation of the manuscript, we found significant text overlap between your submission and previous work in the [introduction, conclusion, etc.].   We would like to make you aware that copying extracts from previous publications, especially outside the methods section, word-for-word is unacceptable. In addition, the reproduction of text from published reports has implications for the copyright that may apply to the publications.   Please revise the manuscript to rephrase the duplicated text, cite your sources, and provide details as to how the current manuscript advances on previous work. Please note that further consideration is dependent on the submission of a manuscript that addresses these concerns about the overlap in text with published work.   [If the overlap is with the authors’ own works: Moreover, upon submission, authors must confirm that the manuscript, or any related manuscript, is not currently under consideration or accepted elsewhere. If related work has been submitted to PLOS ONE or elsewhere, authors must include a copy with the submitted article. Reviewers will be asked to comment on the overlap between related submissions ([http://journals.plos.org/plosone/s/submission-guidelines#loc-related-manuscripts)](http://journals.plos.org/plosone/s/submission-guidelines#loc-related-manuscripts)).]   We will carefully review your manuscript upon resubmission and further consideration of the manuscript is dependent on the text overlap being addressed in full. Please ensure that your revision is thorough as failure to address the concerns to our satisfaction may result in your submission not being considered further | The test overlaps have been amended. The remaining portions are mainly published protocols and statistical analysis. Citations have also been provided. |
| We note that you have stated that you will provide repository information for your data at acceptance. Should your manuscript be accepted for publication, we will hold it until you provide the relevant accession numbers or DOIs necessary to access your data. If you wish to make changes to your Data Availability statement, please describe these changes in your cover letter and we will update your Data Availability statement to reflect the information you provide | The accession numbers have been provided in Supplementary files (Table S2) |

| **Reviewer 1** | **Responses to reviewer’s comments** |
| --- | --- |
| The citation should be changed to square brackets | This has done throughout the document |
| Take care of my inputs in two parts in the attached document. | Amendments done (line 103-104 and 460) |
| Check the reference list to be clear to the journal format. | Ammended as suggested |

| **Reviewer 2** | **Responses to reviewer’s comments** |
| --- | --- |
| Is the bacterial composition and aquatic habitat metabolites in the aquatic habitats affected by seasonality? | Yes, seasonality has been observed to affect bacterial and metabolite composition in aquatic habitats as has been demonstrated by previous studies (Yi et al., 2021). This study was done from Nov-Jan which is generally the end of short rains and the beginning of the dry season in Kenya. Financial constraints could however not allow for more data to be collected to analyse the effects of seasonality. Further studies can be conducted to assess this effect in the irrigated and non- irrigated area |
| Did you assess other factors such as aquatic predators? | Yes, larval and predator densities were also assessed, however, the interest of this research was the microbial and metabolite composition and how they affect larval presence/ absence. Other confounding factors were not analyzed in this study |
| In the irrigation and non-irrigation, what was the major crop? Did you recorded any use of pesticides? That could have impacts to mosquito larvae and water quality? | Agricultural activities have been indicated in Line 104-108  Information on pesticide use in the region is already published in previous research (Orondo et al., 2021) |
| **Sample collection:** | |
| The water samples were pooled into 12 bottle sets and used for microbial analysis. Why 12 bottles? What was the time interval between sample collection and analysis? Was there any preservatives added in water samples? | 12 sets were used. Pools were done every month from Nov 2021 to Jan 2022 (Line112) in both irrigated and non- irrigated area and larval positive and negative habitats. This resulted in 4 pools monthly. Below is a diagrammatic illustration of water sample collection each month    All analysis were done between January- February 2022. The (line 118) indicates how the water samples were stored and preserved awaiting analysis |
| **Major observation:** | |
| The article focus mainly on water sample analysis, the author need to explain how the mosquito larvae were collected, identified, which keys and how were they correlated with bacterial composition and aquatic habitat metabolites | A statement on larval sampling ahs been included in Line 119-121. However, the main focus of this study was larval availability in relation to water and metabolite composition and that is why mosquito densities were not included |
| My suggestion would be add a section describing how mosquito larvae were collected and processed as well. The analysis section should also show how was the analysis of mosquitoes done and how each factor observed influenced abundance/presence of mosquito larvae | Line 119-121 describes larval sampling and morphological identification.  As the main focus of this study was larval availability in relation to water and metabolite composition, further analysis on larvae were not included |
| Map of study area showing irrigated and non-irrigated sites? | A map has been included (Fig1) |
| Analysis section should show how these metabolites affect abundance of mosquito larvae | The study focus (as indicated in the title) was on larval presence/ availability and not larval abundance and that is why mosquito densities were not included |
| **Conclusion:** | |
| “In addition, high fatty acid concentration may have an impact on larval production in both irrigated and non-irrigated areas whereas chlorophyll, sugar, and manganese promoted growth” How did you come up with this conclusion? Please refer to the comment No. 8 above. These should be analysis to assess how this affect larval abundance. Off course a GLMM approach can be used to count for random and fixed variables | This statement (Line 534-537) has been re-phrased to “Finally, high fatty acid concentration may have an impact on larval availability in both irrigated and non-irrigated areas, in addition to chlorophyll, sugar, and manganese promoted larval availability in the aquatic habitats”.  We believe that this is a clearer statement to explain what the study was looking into. |
